# Aldosterone Contributes to Vasopressin Escape through Changes in Water and Urea Transport

**DOI:** 10.3390/biomedicines11071844

**Published:** 2023-06-27

**Authors:** Yanhua Wang, Lauren M. LaRocque, Joseph A. Ruiz, Eva L. Rodriguez, Jeff M. Sands, Janet D. Klein

**Affiliations:** Renal Division, Department of Medicine, Emory University, Atlanta, GA 30322, USA; ywang68@emory.edu (Y.W.); laurenlarocque362@gmail.com (L.M.L.); joseph.ruiz01215@gmail.com (J.A.R.); eva.rodriguez@emory.edu (E.L.R.); janet.klein@emory.edu (J.D.K.)

**Keywords:** aldosterone, mineralocorticoid receptor, phosphatase, calcineurin, hyponatremia, syndrome of inappropriate antidiuretic hormone secretion

## Abstract

Hyponatremia (hypo-osmolality) is a disorder of water homeostasis due to abnormal renal diluting capacity. The body limits the degree to which serum sodium concentration falls through a mechanism called “vasopressin escape”. Vasopressin escape is a process that prevents the continuous decrease in serum sodium concentration even under conditions of sustained high plasma vasopressin levels. Previous reports suggest that aldosterone may be involved in the vasopressin escape mechanism. The abilities of aldosterone synthase (Cyp11b2) knockout and wild-type mice to escape from vasopressin were compared. Wild-type mice escaped while the aldosterone synthase knockout mice did not. Both the water channel aquaporin 2 (AQP2) and the urea transporter UT-A1 protein abundances were higher in aldosterone synthase knockout than in wild-type mice at the end of the escape period. Vasopressin escape was also blunted in rats given spironolactone, a mineralocorticoid receptor blocker. Next, the role of the phosphatase, calcineurin (protein phosphatase 2B, PP2B), in vasopressin escape was studied since aldosterone activates calcineurin in rat cortical collecting ducts. Tacrolimus, a calcineurin inhibitor, blunted vasopressin escape in rats compared with the control rats, increased UT-A1, AQP2, and pS256-AQP2, and decreased pS261-AQP2 protein abundances. Our results indicate that aldosterone regulates vasopressin escape through calcineurin-mediated protein changes in UT-A1 and AQP2.

## 1. Introduction

Hyponatremia (hypo-osmolality) is a disorder of water homeostasis due to abnormal renal diluting capacity. It is the most common clinical electrolyte disorder in hospitalized patients, with an incidence of 15% and a prevalence of 30% [1,2]. Patients with hyponatremia have high levels of plasma vasopressin (antidiuretic hormone, ADH) due to the non-osmotic release of vasopressin, as observed in heart failure, liver disease, and inappropriately high levels of circulating vasopressin in the “syndrome of inappropriate ADH secretion” (SIADH). The continuous secretion of vasopressin increases renal water reabsorption from the kidney collecting duct and urinary sodium loss, thus causing hyponatremia. Although severe hyponatremia can be life-threatening, under most circumstances with inappropriately high plasma vasopressin levels, the body limits the degree to which serum sodium concentration falls through a mechanism called “vasopressin escape” [3]. When vasopressin escape occurs, water excretion increases and urinary osmolality decreases, despite the sustained high levels of vasopressin in the kidney. In the absence of vasopressin escape, decreases in serum sodium concentration can rapidly become life-threatening. These patients can develop headache, lethargy, nausea, reversible ataxia, psychosis, seizures, and even coma due to cerebral edema, which can lead to increased intracerebral pressure, tentorial herniation, respiratory depression, and death. Hyponatremia is associated with as much as a 50% increase in mortality in hospitalized patients [1,2].

Vasopressin, acting through adenylyl cyclase and protein kinase A, phosphorylates the urea transporter UT-A1 and the water channel aquaporin 2 (AQP2), thereby increasing urea and water reabsorption in the kidney [4,5,6]. UT-A1 promotes water reabsorption by increasing osmolality with accumulated urea in the interstitium of the inner medulla [7]. In response to vasopressin stimulation, phosphorylated UT-A1 and AQP2 are transported from subapical intracellular storage vesicles to the apical plasma membrane, thus increasing urea and water permeability in renal collecting duct principal cells. Serine 256 and serine 261 are primary phosphorylation sites on the AQP2 protein. The phosphorylation of serine 256 is reported to promote AQP2 trafficking to the apical plasma membrane [8], while the phosphorylation of serine 261 on AQP2 promotes AQP2 endocytosis from the apical plasma membrane and is associated with ubiquitination and probably proteasomal degradation of AQP2 [9]. Likewise, UT-A1 phosphorylation at serine 499 is essential for inserting UT-A1 into the plasma membrane [10]. Given the critical role of phosphorylation of AQP2 and UT-A1 in stimulating water reabsorption, we examined whether reversing the effect of vasopressin to produce vasopressin escape might involve dephosphorylating UT-A1 and AQP2. There is evidence showing that decreased vasopressin-sensitive AQP2 activity is associated with vasopressin escape [11]. The relevance of AQP2 to vasopressin escape may involve dephosphorylation of AQP2 by specific phosphatases. Our previous findings indicate that the inhibition of protein phosphatases PP2A and PP2B (calcineurin) increases urea and water permeability [12], suggesting that these phosphatases may contribute to the vasopressin escape mechanism.

Previous studies indicate that in animal models of SIADH, the very low serum sodium concentration increases systemic aldosterone [13,14], suggesting that vasopressin escape may be associated with an increase in aldosterone. However, it is unclear how increased aldosterone can limit the action of vasopressin and lead to vasopressin escape. Aldosterone is a steroid hormone that influences water and salt regulation in the renal distal tubule [15,16,17] and the collecting duct [18]. Aldosterone is produced in the adrenal cortex and binds to mineralocorticoid receptors (MRs). This hormone activates calcineurin in rat cortical collecting ducts through a transcription-independent mechanism [19,20]. Inhibition of calcineurin modifies the phosphorylation of UT-A1 and AQP2 in the inner medulla [21] and increases osmotic water permeability in the inner medullary collecting duct [12]. These findings suggest that aldosterone regulates vasopressin escape through a calcineurin-dependent mechanism.

This study provides direct evidence that aldosterone leads to vasopressin escape by regulating AQP2 and UT-A1 activities. We first compared the changes in the protein levels of UT-A1 and AQP2 in aldosterone synthase (Cyp11b2) knockout and wild-type mice during vasopressin escape. We then confirmed aldosterone-dependent vasopressin escape by inhibiting mineralocorticoid receptors (MRs) with spironolactone. We finally examined the effect of inhibition of calcineurin on vasopressin escape and the changes in the total protein and phosphorylation levels of UT-A1 and AQP2 proteins.

## 2. Materials and Methods

### 2.1. Animals

All animal surgical procedures were designed to adhere to the NIH standards for animal use and were approved by the Emory Institutional Animal Care and Use Committee (protocol number “PROTO 201800110, approved 1 September 2020). Both male and female rats (100–140 g) for measuring vasopressin escape were purchased from Charles River Laboratories, Wilmington, MA, USA. The wild-type mice used for these studies were sourced from our colonies maintained at Emory University School of Medicine under the supervision of the Division of Animal Resources. Aldosterone synthase (Cyp11b2) knockout mice were obtained from Dr. James Luther, Vanderbilt University (Nashville, TN, USA) and then maintained in a colony in the Emory animal facilities under supervision of the Emory Division of Animal Resources.

### 2.2. Vasopressin Escape Protocol

We used a well-described rodent model of vasopressin escape [13,14,22] to study the role of aldosterone in vasopressin escape. In this model, animals were placed in metabolic cages overnight to assess basal urine concentration, then implanted with 14-day osmotic minipumps delivering 5 ng/h ddAVP (Desmopressin, Sigma-Aldrich, St. Louis, MO, USA) to promote kidney collecting duct water reabsorption. After urine osmolality increased, indicating a response to vasopressin, the animals were given a gel diet to force additional fluid consumption.

Osmolality was measured in daily 24 h urine collections throughout the experiment using a Wescor vapor pressure osmometer (Logan, UT, USA). In preliminary experiments, serum sodium concentration was measured using a Medica EasyLyte electrolyte analyzer (Bedford, MA, USA) and blood glucose was measured using a LifeScan One Touch Ultra glucometer (Malvern, PA, USA). Sodium concentration correlated with serum osmolality. Blood glucose was normal and did not make a significant contribution to serum osmolality. We did not measure blood urea nitrogen levels in these animals.

Vasopressin escape is indicated by a reduction in urine osmolality despite the continual presence of vasopressin from the osmotic minipump. If urine osmolality remains high, that indicates a failure to escape from vasopressin. At the end of vasopressin escape, animals were sacrificed, and inner medullas (IMs) were dissected from the kidneys for further detection of phosphorylation of AQP2 and UT-A1 proteins.

To test the role of aldosterone, aldosterone synthase (Cyp11b2) knockout and wild-type litter-mate mice were placed into the vasopressin escape protocol. Aldosterone synthase (Cyp11b2) knockout mice fail to produce aldosterone [23]. Mice were sacrificed and protein lysates were prepared for Western blot analysis of total and phosphorylated AQP2 and total and phosphorylated UT-A1. To confirm our observations from the aldosterone synthase knockout mice, rats were placed into the vasopressin escape protocol described above and given the mineralocorticoid receptor (MR) blocker spironolactone (200 µL/d, s.c., Sigma-Aldrich, St. Louis, MO, USA), or vehicle.

To test whether calcineurin regulates AQP2 and UT-A1 during vasopressin escape, rats were placed into the vasopressin escape protocol. After 4 days of increased osmolality, all rats were given a gel diet to force fluid consumption, and half received daily injections of 1 mg/kg/day tacrolimus (Sigma-Aldrich, St. Louis, MO, USA) for a further 4 days. Rats were sacrificed and protein lysates were prepared for Western blot analysis of total and phosphorylated AQP2 protein and total and phosphorylated UT-A1 protein.

### 2.3. Western Blot Analysis

Inner medullary protein lysate at a concentration of 1 mg/mL was loaded (20 µg/lane) into wells of polyacrylamide gels with each well containing a different animal’s lysate. Proteins were size separated on 12.5% gels for AQP2 or 10% gels for UT-A1, then electroblotted to polyvinylidene difluoride membranes (PVDF Immobilon-P, EMD-MilliporeSigma, Burlington, MA, USA). Membranes were bathed in a 5% solution of Carnation nonfat dry milk in TBS (Tris-buffered saline: 0.5 M NaCl, 20 mM Tris-HCl, pH 7.5) to block open sites. Primary antibodies were prepared in the 5% milk/TBS buffer with 0.1% Tween 20 added. Blots were incubated with primary antibodies at 4 °C overnight. All primary antibodies were prepared in rabbit. Alexa Fluor 680-linked antirabbit IgG (1:4000 dilution) (Molecular Probes, Eugene, OR, USA) was used as the secondary antibody to identify bound primary antibodies. The Alexa fluor was detected using the LICOR Odyssey protein analysis system (Lincoln, NE, USA). Antibodies to phosphorylated AQP2 p-serine 256 or p-serine 261 (1:2000 dilution) were purchased from Cell Signaling Technology, Danvers, MA, USA and used at a dilution of 1:2000. Antibodies to AQP2 (1:2000) or UT-A1 (1:1000) were made in our laboratory [24]. Antibodies to p-serine 499-UT-A1 (1:500 dilution) were purchased from PhosphoSolutions, Aurora, CO, USA. Ponceau staining (Sigma-Aldrich, St. Louis, MO, USA) of the whole gel was used as a loading control.

### 2.4. Statistics

All data are presented as mean ± s.e. Urine osmolalities from vasopressin escape studies were analyzed using a longitudinal analysis. The data from the protein analysis studies were analyzed using a Student’s *t*-test for two groups. The criterion for statistical significance is *p* < 0.05.

## 3. Results

### 3.1. Vasopressin Escape Was Observed in Wild-Type but Not in Aldosterone Synthase Knockout Mice

**Figure 1:**
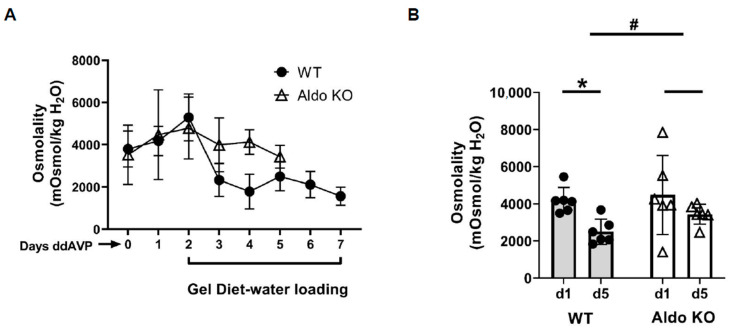
Litter-mate wild-type mice (WT) escape from vasopressin while aldosterone synthase knockout (KO) mice do not. (**A**) 24 h urine osmolality measured daily after providing the gel diet to initiate vasopressin escape in wild-type (WT, closed circles) and aldosterone synthase (Aldo KO, open triangles) mice. (**B**) Bar graph showing the urine osmolalities on day 1 and day 5 of the escape period in WT and Aldo KO mice. Data: mean ± s.e., *n* = 6, * = *p* < 0.05 WT vs. WT escape, # = longitudinal statistical analysis of the overall effect of the treatment on the change in urine osmolality between the WT and Aldo KO mice.

To determine if aldosterone mediates vasopressin escape, aldosterone synthase (Cyp11b2) knockout and wild-type litter-mate mice were implanted with 14-day osmotic minipumps delivering 5 ng/h ddAVP (Desmopressin, a selective type 2 vasopressin receptor (V2R) agonist). After 2 days of increased urine osmolality, mice were given a gel diet to force additional fluid consumption. Urine osmolality of the wild-type mice was significantly decreased from 4181 ± 283 to 2499 ± 277 mosmol/kg H_2_O (*n* = 6, *p* < 0.05; Figure 1).

**Figure 2:**
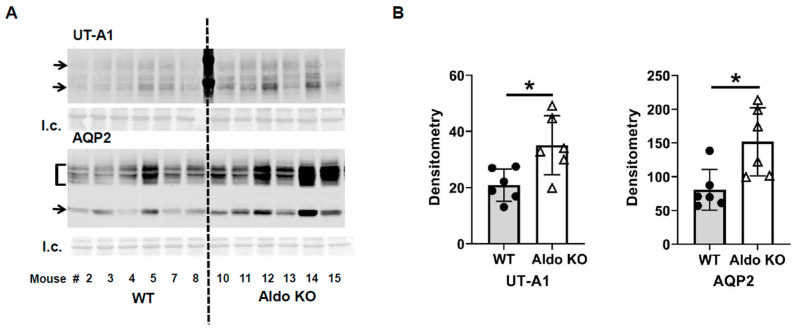
Aldosterone synthase knockout mouse levels of UT-A1 and AQP2 proteins at the end of the vasopressin escape protocol. (**A**) Western blots of kidney inner medullas from WT and Aldo KO mice probed for UT-A1 and AQP2 proteins. Arrows/brackets denote molecular weights of proteins (UT-A1: 117, 97 kDa; AQP2: 35–45, 29 kDa). Below each Western blot is the total protein loading control (l.c.). Each lane is a sample from a different rat collected at the same time in the escape protocol. (**B**) Average density (arbitrary units) of protein bands ± s.e., *n* = 6/group, closed circles: total UT-A1, open triangles: pSer499-UT-A1, * = *p* < 0.05 vs. WT.

In contrast, the urine osmolality of the aldosterone synthase knockout mice was only slightly reduced from 4479 ± 868 to 3435 ± 221 mosmol/kg H_2_O (*n* = 6; Figure 1). The change in urine osmolality between day 5 and day 1 in wild-type mice was significantly different from the change in knockout mice (*p* < 0.05, Figure 1). Western blots of inner medullary (IM) tissue lysates were probed for AQP2 and UT-A1 proteins at the end of the vasopressin escape period. Analysis of protein abundances showed that both AQP2 and UT-A1 proteins were higher in the aldosterone synthase knockout mice than in the wild-type mice at the end of the escape period (AQP2: 57 ± 12 (WT mice) vs. 178 ± 44 (aldosterone synthase knockout mice), *n* = 6, *p* < 0.05; UT-A1: 47 ± 9 (WT mice) vs. 76 ± 14 (aldosterone synthase knockout mice), *n* = 6, *p* < 0.05; Figure 2).

### 3.2. Inhibition of the Mineralocorticoid Receptor (MR) Blunted Vasopressin Escape

**Figure 3:**
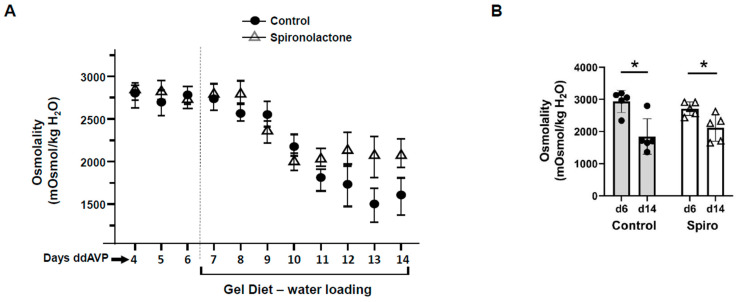
Blunting of vasopressin escape by blocking mineralocorticoid receptors with spironolactone in rats. (**A**) Urine osmolality values daily for the escape protocol. Gel diet initiated after day 6. (**B**) Bar graph showing the urine osmolalities on day 6 and day 14 of the vasopressin escape period in rats treated with or without spironolactone (Spiro). Bars = mean ± s.e., *n* = 5, * *p* < 0.05 vs. control (d6).

To confirm that aldosterone regulates vasopressin escape, spironolactone was used to block MRs in rats. After 6 days of increased urine osmolality, rats were given a gel diet to force additional fluid consumption. The urine osmolality of vehicle-treated rats and spironolactone-treated rats was decreased from 2938 ± 154 to 1846 ± 248 mosmol/kg H_2_O and from 2710 ± 93 to 2112 ± 188 mosmol/kg H_2_O, respectively (*n* = 5, *p* < 0.05; Figure 3). The change in urine osmolality in the spironolactone-treated rats was reduced compared with that in the vehicle-treated rats, but not significantly (Figure 3). The spironolactone-treated rats developed severe hyponatremia: serum sodium concentration was 113 ± 5 mEq/l in spironolactone-treated rats compared with 131 ± 3 mEq/l in vehicle-treated rats (*n* = 5, *p* < 0.02).

### 3.3. Inhibition of Calcineurin Diminished Vasopressin Escape

**Figure 4:**
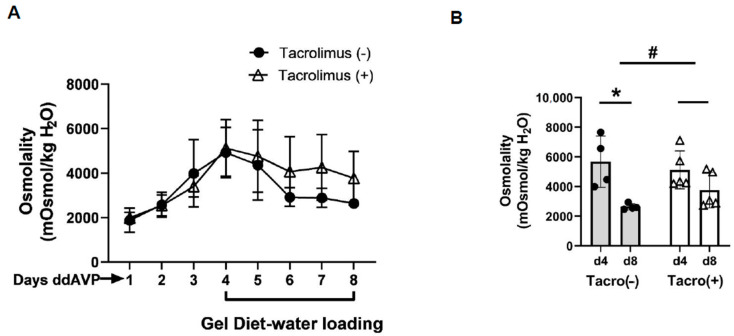
Evidence for vasopressin escape and its prevention by tacrolimus. (**A**) Urine osmolality values daily for the escape protocol. The gel diet was initiated after day 4. ddAVP = vasopressin. (**B**) Bar graph showing the urine osmolalities on day 4 and day 8 of the escape period in rats treated with or without tacrolimus (Tacro). Data: mean ± s.e., *n* = 4, * = *p* < 0.05, # = longitudinal statistical analysis of the overall effect of the treatment on the change in urine osmolality between vehicle-treated rats (Tacro (−)) and the Tacro-treated (Tacro (+)) rats.

Aldosterone activates calcineurin in rat cortical collecting ducts [19,20]. To determine the role of calcineurin in vasopressin escape, tacrolimus was used to inhibit calcineurin. After 4 days of increased urine osmolality, rats were given a gel diet to force additional fluid consumption. The urine osmolality dropped from 5126 ± 572 to 3768 ± 539 mosmol/kg H_2_O in the tacrolimus-treated rats while it dropped from 5675 ± 866 to 2638 ± 104 mosmol/kg H_2_O in the vehicle-treated rats (*n* = 4, *p* < 0.05, Figure 4). The change in urine osmolality between day 5 and day 1 in the vehicle-treated rats was significantly different from that in the tacrolimus-treated rats (*p* < 0.05, Figure 4).

### 3.4. Inhibition of Calcineurin Increased UT-A1 and AQP2 Protein Abundances during Vasopressin Escape

**Figure 5:**
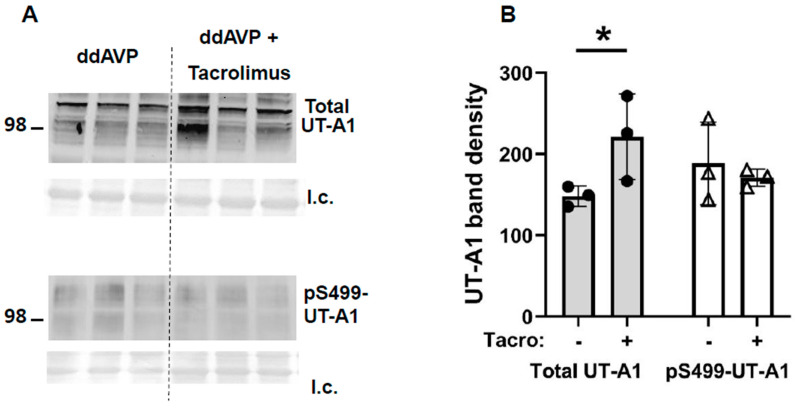
In inner medullas of rats undergoing the vasopressin escape protocol, tacrolimus increased total UT-A1 but not pSer499-UT-A1 protein. (**A**) Representative Western blots showing total UT-A1 (top) and pSer499-UT-A1 (bottom) abundances in rat IM with control (ddAVP) vs. tacrolimus for 30 min. Below each Western blot is the total protein loading control (l.c.). Each lane is a sample from a different rat collected at the same time in the escape protocol. (**B**) The bar graph shows the average of the combined density of both UT-A1 glycoprotein protein forms (117 and 97 kDa) (arbitrary units) ± s.e., *n* = 4–6/group, closed circles: total UT-A1, open triangles: pSer499-UT-A1, * = *p* < 0.05 vs. WT.

Inner medullary tissue lysates were analyzed using Western blot analysis, probing for UT-A1 or AQP2 proteins at the end of the vasopressin escape period. Tacrolimus increased total UT-A1 protein (vehicle-treated: 148 ± 7, tacrolimus-treated: 221 ± 30, *n* = 4–6, *p* < 0.05, Figure 5) but did not increase pS499-UT-A1 (vehicle-treated: 188 ± 29, tacrolimus-treated: 171 ± 6, *n* = 4–6, Figure 5). Figure 5 shows the Western blots of representative samples from three different animals per group collected at the same time in the escape protocol.

**Figure 6:**
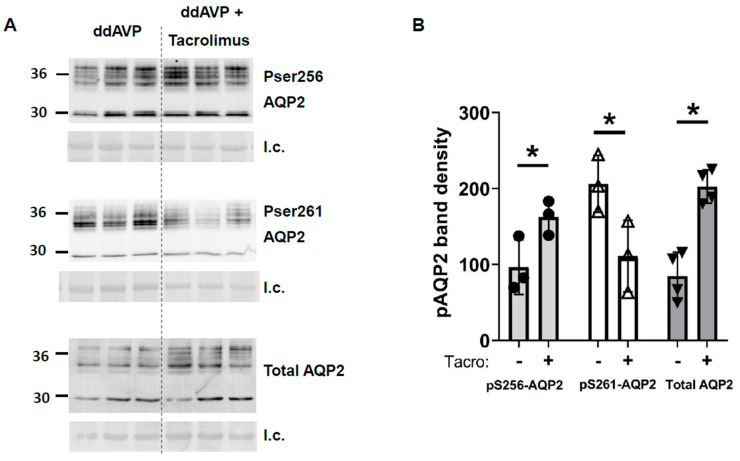
In inner medullas of rats undergoing the vasopressin escape protocol, tacrolimus increased total AQP2 and pS256-AQP2, and decreased pS261-AQP2 protein. (**A**) Western blots of kidney inner medullas from control and tacrolimus-treated rats probed for AQP2, pS256 AQP2, and pS261 AQP2. Molecular weights of AQP2: 35–45, 29 kDa. Below each Western blot is the total protein loading control (l.c.). Each lane is a sample from a different rat collected at the same time in the escape protocol. (**B**) Bars = average density of combined glycosylated + unglycosylated bands ± s.e., *n* = 3–4, closed circles: pS256 AQP2, open triangles: pS261 AQP2, closed triangles: total AQP2; * = *p* < 0.05.

Next, we measured AQP2 protein. Phospho-Ser256-AQP2 was increased (vehicle-treated: 97 ± 21, tacrolimus-treated: 163 ± 13, *n* = 3, *p* < 0.05, Figure 6), pSer261-AQP2 was decreased (vehicle-treated: 206 ± 22, tacrolimus-treated: 111 ± 27, *n* = 3, Figure 6), and total AQP2 was increased by tacrolimus (vehicle-treated: 85 ± 16, tacrolimus-treated: 203 ± 11, *n* = 4, Figure 6).

## 4. Discussion

Vasopressin escape is the body’s defense mechanism to limit the degree of hyponatremia (hypo-osmolality) through a process that counters the water-retaining action of vasopressin [3]. Increased urine volume and decreased urine osmolality are hallmark physiological parameters indicative of vasopressin escape. Previous studies show that aldosterone is associated with vasopressin escape because of its ability to regulate renal sodium transport [13,14]. However, a limited number of studies have been performed to explain the change in AQP2 or water transport, which is the primary mechanism underlying vasopressin escape. The movement of urea from the tubular lumen to the medullary interstitium is crucial to the increase in medullary hypertonicity that provides a driving force for water movement within the kidney. This makes urea transport integral to water movement [25]. Therefore, understanding how aldosterone affects UT-A1 is also important for comprehending its role in vasopressin escape. This study provides evidence that aldosterone regulates vasopressin escape by mediating the dephosphorylation of AQP2 and UT-A1.

Nielsen’s group showed that aldosterone reduces whole cell (cortical collecting duct principal cell) AQP2 expression in the presence of desmopressin acetate (ddAVP) [26]. When infused with aldosterone, normal rats and rats with diabetes insipidus developed polyuria with reduced urine osmolality [27]. These studies suggest aldosterone’s ability to counteract the water retention caused by high levels of vasopressin. To verify the role of aldosterone in vasopressin escape, aldosterone synthase knockout and wild-type litter-mate mice were placed into the vasopressin escape protocol [13,14,22]. The wild-type mice displayed a significantly decreased urine osmolality, while the aldosterone synthase knockout mice did not (Figure 1), indicating that vasopressin escape occurred in the wild-type mice, but failed in the aldosterone synthase knockout mice. We also assessed the abundance of AQP2 and UT-A1 in inner medullary tissue lysates from these mice. The aldosterone synthase knockout mice exhibited a higher protein abundance of AQP2 and UT-A1 than the wild-type mice at the end of the escape period (Figure 2), indicating that lack of aldosterone promotes AQP2 and UT-A1 expression during vasopressin escape, which supports the concept from Nielsen’s group that aldosterone reduces whole cell AQP2 expression in combination with ddAVP. Our results suggest that aldosterone plays an important role in vasopressin escape, and that AQP2 and UT-A1 may be involved in aldosterone’s mediation of vasopressin escape. This conclusion was further supported by the finding of a blunted decrease in urine osmolality in spironolactone-treated rats during the vasopressin escape protocol (Figure 3). Spironolactone is an MR blocker. By interrupting the interaction of aldosterone and its receptors, the spironolactone-treated rats maintained a higher urine osmolality compared with the vehicle-treated rats, thus developing severe hyponatremia (low serum sodium concentration). However, although spironolactone impeded vasopressin escape, its inhibitory role was not statistically significant, which may be attributed to inefficient absorption of spironolactone in the animals or incomplete inhibition of the MR, in contrast to the complete absence of aldosterone in the aldosterone synthase knockout mice. We did not measure cortisol in the aldosterone synthase knockout animals so we cannot exclude the possibility that the decrease in vasopressin escape in the knockout mice could be influenced by the glucocorticoid action on the mineralocorticoid receptor. If this was contributing to the vasopressin escape process, then the effect of aldosterone would be even more profound.

Aldosterone is a mineralocorticoid hormone that maintains water and electrolyte balance by modulating sodium channels and sodium transport. Aldosterone interacts with its intracellular MR, and the combination of aldosterone and MRs activates genomic pathways to control sodium channel and sodium transporter gene transcription [15,16]. The mineralocorticoid receptor and aldosterone have been studied in relation to most of the sodium transporters, including the epithelial sodium transporter (ENaC), the sodium chloride cotransporter (NCC), and sodium potassium ATPase (NaKATPase), as well as potassium transporters such as the renal outer medullary potassium channel (ROMK), the big potassium channel (BK), and their regulatory proteins, such as WNK1, WNK4, Nedd4-2, and others. The products of these genes eventually alter the activity of ionic transport systems located in the apical and the basolateral plasma membranes of kidney epithelial cells by altering the levels of the proteins available for channel and transport activity. However, aldosterone can affect sodium channel and sodium transporter activities through nongenomic mechanisms that involve post-translational modifications of the transporter or channel proteins [28,29]. Calcineurin is a prevalent phosphatase in most cells in the kidney that is responsive to aldosterone. Calcineurin is activated by aldosterone independent from transcription or translation. This allows for a rapid regulatory effect on vasopressin-stimulated osmotic water reabsorption in the collecting duct [19,20]. We previously confirmed the role of calcineurin in aldosterone-regulated water reabsorption [12]. We further tested the effect of calcineurin inhibition on vasopressin escape. Our results indicate that the degree to which urine osmolality dropped in the tacrolimus-treated rats was less than that in the vehicle-treated rats, suggesting that inhibition of calcineurin with tacrolimus prevented vasopressin escape (Figure 4).

Tacrolimus is a widely used antirejection medication that inhibits calcineurin, which is also named phosphatase 2B (PP2B). The mechanism by which hyponatremia results from tacrolimus is unknown. Proposed mechanisms include SIADH and aldosterone resistance. The reduced vasopressin escape in tacrolimus-treated rats can be attributed to the increase in osmotic water permeability in the collecting duct resulting from the higher phosphorylation state of AQP2 when calcineurin is inhibited by tacrolimus [12]. Tacrolimus increased total UT-A1 protein but did not increase pS499-UT-A1 (Figure 5). Phospho-S256-AQP2 and total AQP2 were increased and pS261-AQP2 was decreased by tacrolimus (Figure 6). Both changes (Figure 5 and Figure 6) contribute to an increase in water reabsorption and thereby prevent vasopressin escape; the increase in UT-A1 increases urea reabsorption and inner medullary interstitial osmolality, which builds an osmotic gradient for water transport, and the increase in total AQP2 and S256-AQP2 promotes AQP2 accumulation in the apical plasma membrane, thereby increasing water reabsorption. The phosphorylation of S261 was decreased, suggesting reduced endocytosis of AQP2, thereby increasing AQP2 accumulation in the apical plasma membrane.

It is worth mentioning that calcineurin inhibition with tacrolimus attenuated vasopressin escape in rats (Figure 4) but did not completely prevent vasopressin escape, which implies that there remains some activity that allows for a partial response, which may be due to phosphatases other than calcineurin. Other protein phosphatases such as PP2A that are involved in the regulation of water reabsorption [12] may be a supplemental mechanism that contributes to vasopressin escape in addition to aldosterone activation of calcineurin. Future studies are needed to identify the remaining phosphatases that contribute to vasopressin escape.

In this study, we analyzed the changes in urine osmolality during vasopressin escape using a longitudinal analysis. A longitudinal analysis allows one to observe the changes in a variable over a period of time, during which repeated measurements for the variable on the same individuals are performed. We observed urine osmolalities for 14 days by measuring the urine osmolality of each animal on each experimental day. Therefore, our osmolality data were subject to a longitudinal analysis. A longitudinal analysis takes into account the values at all time points and provides an inference for the effect of treatment.

## 5. Conclusions

In conclusion, the present study shows that aldosterone acts through calcineurin to mediate vasopressin escape. Vasopressin escape is prevented if aldosterone or its receptor is not available, or calcineurin is inhibited. Increased protein abundance of AQP2 or UT-A1, or pS256-AQP2, and decreased expression of pS261-AQP2 contribute to deficient vasopressin escape in the absence of aldosterone. This study provides a new understanding of vasopressin escape in terms of phosphatase-regulated water channel activities, suggesting a new direction for therapies for hyponatremia.

## Data Availability

Not applicable.
